# Using the Internet to Understand Smokers’  Treatment Preferences: Informing Strategies to Increase Demand

**DOI:** 10.2196/jmir.1666

**Published:** 2011-08-26

**Authors:** J Lee Westmaas, Lorien Abroms, Jeuneviette Bontemps-Jones, Joseph E Bauer, Jeanine Bade

**Affiliations:** ^1^Behavioral Research CenterAmerican Cancer SocietyAtlanta, GAUnited States; ^2^Behavioral Sciences and Health EducationRollins School of Public HealthEmory UniversityAtlanta, GAUnited States; ^3^Department of Prevention and Community HealthSchool of Public Health and Health ServicesGeorge Washington UniversityWashington, DCUnited States; ^4^Statistics and Evaluation CenterAmerican Cancer SocietyAtlanta, GAUnited States; ^5^Department of Health PromotionsAmerican Cancer SocietyAtlanta, GAUnited States

**Keywords:** Consumer demand, pharmacotherapy, quitline counseling, smoking cessation, social support

## Abstract

**Background:**

Most smokers attempt to quit on their own even though cessation aids can substantially increase their chances of success. Millions of smokers seek cessation advice on the Internet, so using it to promote cessation products and services is one strategy for increasing demand for treatments. Little is known, however, about what cessation aids these smokers would find most appealing or what predicts their preferences (eg, age, level of dependence, or timing of quit date).

**Objective:**

The objective of our study was to gain insight into how Internet seekers of cessation information make judgments about their preferences for treatments, and to identify sociodemographic and other predictors of preferences.

**Methods:**

An online survey assessing interest in 9 evidence-based cessation products and services was voluntarily completed by 1196 smokers who visited the American Cancer Society’s Great American Smokeout (GASO) webpage. Cluster analysis was conducted on ratings of interest.

**Results:**

In total, 48% (572/1196) of respondents were “quite a bit” or “very much” interested in nicotine replacement therapy (NRT), 45% (534/1196) in a website that provides customized quitting advice, and 37% (447/1196) in prescription medications. Only 11.5% (138/1196) indicated similar interest in quitlines, and 17% (208/1196) in receiving customized text messages. Hierarchical agglomerative cluster analysis revealed that interest in treatments formed 3 clusters: *interpersonal* *–* *supportive* methods (eg, telephone counseling, Web-based peer support, and in-person group programs), *nonsocial* *–* *informational* methods (eg, Internet programs, tailored emails, and informational booklets), and *pharmacotherapy* (NRT, bupropion, and varenicline). Only 5% (60/1196) of smokers were “quite a bit” or “very much” interested in interpersonal–supportive methods compared with 25% (298/1196) for nonsocial–informational methods and 33% (399/1196) for pharmacotherapy. Multivariate analyses and follow-up comparisons indicated that level of interest in pharmacotherapy (“quite a bit or “very much” vs. “not at all”) varied as a function of education (n = 575, χ^2^_3_ =16.6, *P* = .001), age (n = 528, χ^2^_3_ = 8.2, *P* = .04), smoking level (n = 514, χ^2^_3_ = 9.5, *P* = .02), and when smokers were planning to quit (n = 607, χ^2^_4_ = 34.0, *P* < .001). Surprisingly, greater age was associated with stronger interest in nonsocial–informational methods (n = 367, χ^2^_3_ = 10.8, *P* = .01). Interest in interpersonal–supportive methods was greater if smokers had used a quitline before (n = 259, χ^2^_1_ = 18.3, *P* < .001), or were planning to quit earlier rather than later (n = 148, χ^2^_1_ = 4.9, *P* = .03).

**Conclusions:**

Smokers accessing the Internet for information on quitting appear to differentiate cessation treatments by how much interpersonal interaction or support the treatment entails. Quitting date, smoking level, and sociodemographic variables can identify smokers with varying levels of interest in the 3 classes of cessation methods identified. These results can potentially be used to more effectively target and increase demand for these treatments among smokers searching the Internet for cessation information.

## Introduction

Several effective tobacco-cessation products and services are available to help smokers who want to quit. These can double or triple the rate of cessation compared with quitting without help [1]. In spite of their availability, however, the use of these products and services is low, as most smokers opt to quit on their own [2]. For example, the North American Quitline Consortium estimated that the reach of quitlines, calculated as the proportion of all smokers in a US state who complete a program of phone counseling, was between only 1% and 2% [3].

There has been little research on why smokers are not using evidenced-based treatments to quit. One study found that, although smokers planning to quit expressed several barriers around quitline use, none of the self-reported barriers predicted actual calls made to a quitline [4]. Another study of 636 German smokers who had never used cessation aids when trying to quit found that the most endorsed barrier to not using cessation aids was belief in one’s own ability to quit [5]. A quarter of smokers also believed that cessation aids would not be helpful, and cited their cost as a reason for not using them. Lack of awareness of cessation aids also appears to be a barrier. In a study of smokers in the United Kingdom, only 30% of current and former smokers were aware of various cessation services provided by the National Health Service [6]. This is consistent with findings from a study of Canadian smokers, who demonstrated low recall of cessation methods [7].

To increase utilization of cessation products and services, the National Tobacco Cessation Collaborative, an American and Canadian consortium of leading nonprofit and government agencies dedicated to reducing the burden of tobacco use, delineated several core strategies to increase demand for available treatments. One of these strategies includes the recommendation to understand what smokers need and want, instead of viewing them as “passive treatment beneficiaries rather than treatment consumers” (p. S308) [8]. The millions of smokers who seek cessation information on the Internet [9] are a population that can be targeted to increase demand for treatments. The Internet can also be used to understand their cessation needs and wants, and to easily provide access to the treatments they prefer. However, little is known about preferences for cessation methods in this population. To that end we asked smokers who were seeking cessation information on the Internet to rate their interest in various evidenced-based cessation products and services. We subjected these ratings to a cluster analysis, an analytic technique used extensively by market researchers to evaluate brand or product preferences [10]. We used this approach to determine whether particular groups of products and services were preferred over others so that we could infer underlying motivations for preferences. We also examined whether sociodemographic and smoking behavior variables were associated with preferences for particular treatments. Knowledge of who is interested in what kinds of treatments can be used to promote cessation treatments on the Internet in a more targeted manner, and could potentially increase demand for them.

### Using the Internet to Inform Smokers About Cessation Treatments

Many smokers thinking of quitting access the Internet for general information on smoking cessation. The Internet may therefore be an effective medium for promoting evidenced-based cessation aids [11]. According to a random-digit-dial survey conducted in the United States in 2006, an estimated 9% of Internet users (approximately 12.7 million individuals) searched the Internet for information on “how to quit smoking” [9]. Moreover, compared with traditional media such as radio or television advertising, online advertising has been shown to be a more cost-efficient mode of recruiting smokers to Internet- and telephone-based cessation treatments [12]. It is now also possible for marketers to construct a sociodemographic profile for a computer user by gathering information on different websites visited. This information is then used to market products to groups of people who would be receptive to messages about the products. This strategy, termed “behavioral targeting,” could be employed to promote specific cessation products or services to smokers most likely to use them [13]. In addition, some products and services (eg, Internet programs for cessation) can be made immediately available—that is, at the time smokers are searching the Internet for help on quitting. Overall, these developments speak to the tremendous potential of the Internet as an ideal medium through which large numbers of smokers can be reached and provided with information on, or access to, various evidence-based cessation treatments.

Identifying and targeting potential quitters by providing them information on or immediate access to treatments would be maximally effective if smokers’ preferences for treatments and predictors of those preferences were known. Only 1 study to our knowledge, however, asked smokers seeking cessation information on the Internet to rate their perceptions of various treatments [11]. Results indicated that smokers perceived that telephone counseling or receiving support through Internet chat, forums, or email would be least helpful. In contrast, smokers perceived that information about withdrawal or individually tailored information would be most helpful [11].

Research on what methods are used most frequently by smokers when attempting to quit could provide some indication of what cessation products and services would be favored. Low use or nonuse of a particular product or service, however, may be due to a smoker simply not knowing it exists. Some products may also be used only because they were recommended or were available at the time. Nevertheless, data on actual use of evidence-based cessation treatments can be used to form hypotheses about what types of treatments might be preferred, or how preferences might vary as a function of sociodemographic characteristics. For example, in the 2005 National Health Interview Survey (NHIS) [14], pharmacotherapy was used more frequently than behavioral methods, which was used infrequently (<5%). Results also indicated that older smokers (aged ≥25 years) were more likely than younger smokers to use pharmacotherapy. In addition, for younger smokers, but not for older smokers, greater educational attainment was associated with having used pharmacotherapy. Based on these results we hypothesized that pharmacotherapy and behavioral methods would form separate clusters of preferences, and that interest in pharmacotherapy would be greater than interest in behavioral methods. We also hypothesized that, after controlling for age, greater educational attainment would be associated with greater interest in pharmacotherapy (as more educated smokers are likely to be more knowledgeable about nicotine replacement therapy [NRT] and less susceptible to myths about it).

We also expected preferences to vary by gender. In the 2-year longitudinal National Youth Smoking Cessation Survey of smokers aged 16–24 years [15], among those who had tried to quit smoking at least once, seeking help from a professional was more common among females than among males. The proportion of female smokers who contact quitlines is also greater [16]. This may be a result of the male stereotype emphasizing independence and avoidance of emotional disclosure, or the greater acceptability for females to seek others’ assistance [17]. Based on these results we hypothesized that female smokers would be more interested than male smokers in methods involving counseling or seeking advice from others. In sum, our hypotheses, based on the findings described earlier, were that (1) pharmacotherapy and behavioral methods would form separate clusters, and interest in pharmacotherapy would be greater than interest in behavioral methods, (2) more educated smokers would be more interested than less educated smokers in using pharmacotherapy, and (3) compared with male smokers, female smokers would be more interested in methods involving counseling such as quitlines or group cessation programs. Because of younger smokers’ greater use of the Internet [18] we also wanted to explore the following hypothesis: younger smokers seeking cessation information on the Internet would be more interested than older smokers in using Internet programs for cessation, or other technologically involved treatments (eg, text messages).

## Methods

### Participants

Participants’ mean age was 38.4 years (SD 9.1) and they smoked an average of 15.9 cigarettes per day (SD 9.1). The majority of the sample was female (840, 74.3%). A college degree or higher was reported by 34.2% (388), with 39.6% (449) reporting some college, 21.1% (239) completing high school or its equivalent, and 5.1% (58) completing grade 11 or lower. A minority (43, 3.8%) were Latino/Hispanic, whereas the majority were not (1068, 94.8%), or indicated “Don’t know” (15, 1.4%). The majority of participants were white (984, 87%). Black or African American smokers constituted 5% (57) and Asian smokers, 1.6% (18) of the sample. The remaining racial groups were collapsed into 1 category that comprised 6.4% (75) of the sample (ie, Pacific Islander, American Indian, Alaska Native, Other, and Don’t know) (see Table 1).

**Table 1:** Descriptive statistics of the study population

Characteristic		n	%
**Smoking rate (cigarettes per day)**			
	<10	230	22.1
	10–19	379	36.4
	20–29	329	31.6
	≥30	103	9.9
**Age group (years)**			
	≤25	164	15.7
	26–40	438	41.9
	41–55	358	34.2
	≥56	86	8.2
**Gender**			
	Female	840	74.3
	Male	291	25.7
**Education level**			
	Grade ≤11	58	5.1
	High school graduate or GED^a^	239	21.1
	Some college	449	39.6
	College graduate or higher	388	34.2
**Race**			
	White	984	87.0
	Black/African American	57	5.0
	Asian	18	1.6
	Pacific Islander/American Indian/Alaskan Native/Other/Don’t know	72	6.4
**Quit in past year**			
	Yes	730	63.2
	No	425	36.8
**Quit date**			
	In the next 24 hours	266	22.2
	In next week or two	362	30.3
	In next month	209	17.5
	In next 6 months	114	9.5
	In future/undecided	245	20.5
**Free quitline help available?**			
	Yes	256	21.4
	No	35	3.0
	Don’t know	861	74.7

**Ever used a quitline**			
	Yes	112	9.7
	No	884	76.6
	N/A^b^—never tried to quit before	158	13.7

^a^ General equivalency diploma.

^b^ Not applicable.

### Procedure

A 12-item online questionnaire was posted for 11 months on the American Cancer Society’s (ACS) Great American Smokeout (GASO) webpage. The survey was posted 1 week prior to the 2008 GASO event (November 20). The GASO webpage is the ACS’s online portal for information about quitting and received 92,946 unique views during the study period. An introductory paragraph on the GASO webpage explained that the ACS was interested in learning about how smokers quit, and that if they were interested to click on the link appearing below. The link led respondents to a page in which a consent section appeared above the survey. The survey was completed by 1594 current smokers over the entire study period. Approximately half of responses (845, 53%) were collected by 6 days after GASO, and the other half during the remaining months of the study period. We excluded 90 participants due to missing data on sociodemographic variables. Analyses reported below were restricted to participants who provided responses on sociodemographic variables and who did not choose the “don’t know” option on the items assessing interest in cessation products and services (N = 1196). The voluntary and anonymous survey, which was approved by the Emory University institutional review board, did not provide incentives, financial or otherwise, for completion.

The current study also subjected ratings of interest in cessation products and services to a cluster analysis. Cluster analysis is an assumption-free classification technique that is commonly used in market research to understand consumer behavior [10]. It simultaneously minimizes within-group variance and maximizes between-groups variance, so that members or variables of the same group are more similar to each other than to those of other groups [19]. It can thus be used to infer the underlying dimensions that form the basis of smokers’ preferences among treatments. For the present report we used SPSS 18 (IBM Corporation, Somers, NY, USA) to perform hierarchical agglomerative clustering, with between-groups linkage and squared euclidean distance as the similarity metric. To determine the reliability of clusters obtained we first conducted a cluster analysis on a random sample of 50% of cases (598) and a second one on the remaining cases. For both samples membership in clusters at each level of agglomeration was identical. The composition of clusters was also the same when analyses were conducted on the first 50% and the latter 50% of cases. The final cluster analysis presented thus used the full sample.

### Predictors of Cessation Methods

To examine the relationship between interest in cessation products and services, and sociodemographic and smoking behavior variables, a multivariate analysis of variance (MANOVA) was conducted. Cessation methods that formed clusters were averaged to obtain summary variables that were the set of dependent variables in the MANOVA. Independent variables (categorical) were gender, age, race, education, when smokers were planning to quit, prior use of a quitline, smoking rate, knowledge of quitlines’ free availability, and whether an attempt to quit was made in the past year. A second MANOVA was conducted that excluded independent variables with nonsignificant multivariate main effects (gender, quitline knowledge, and past-year attempt). Multivariate results from this final MANOVA are presented. Analyses that controlled for the number of days between when the survey was completed and the start date of the survey were also conducted.

Follow-up Tukey pairwise comparisons that controlled for the familywise error rate were conducted. These examined differences on dependent variables among levels of the significant independent variables (ie, significant according to the univariate results). For comparisons that the Tukey tests indicated were statistically significant (ie, *P* < .05) we also conducted chi-square tests to demonstrate the association between the independent and dependent variables. For these chi-square analyses we first created 2 contrasting groups for each dependent variable: smokers who were “not at all” interested versus smokers who were “quite a bit” or “very much” interested. Smokers who indicate they are “quite a bit” or “very much” interested in a cessation method are likely to be most receptive to trying a cessation method if it is available to them, at least much more so than smokers who report that they are “not at all” interested. These latter smokers are likely to be more difficult to reach with marketing efforts aimed at encouraging use of a particular cessation method. The chi-square follow-up analyses thus aided interpretation by illustrating which groups might be fairly easy or more challenging to encourage to use particular cessation methods. We followed a conservative approach of reporting only the chi-square associations that were also significant (in addition to the Tukey tests described above). We also provide an effect size measure, Cramer’s V, that ranges from 0 to 1 [20,21].

### Measures

#### Sociodemographic and Smoking Behavior Variables

Participants were asked to indicate when they planned to quit (in the next 24 hours, in the next week or two, in the next month, in the next 6 months, sometime in the future but haven’t decided when, not applicable (N/A)—already quit, other), whether they knew if “free help from a counselor at a quitline” was available to all smokers in their state (yes, no, don’t know), whether they had ever called a quitline to help them quit smoking (yes, no, N/A—never tried to quit before), the number of cigarettes smoked per day, whether they tried to seriously quit in the past year (yes, no), their gender, and their age.

#### Interest in Cessation Products and Services

Smokers were asked to indicate how interested they would be in using the following cessation products and services on a scale from 1 (not at all) to 5 (very much). Specific items, as written, were (1) “Using a telephone quitline (a quitline has trained counselors help you over the phone with your quit attempt,” (2) “Using a website that gives professional advice about quitting smoking that is customized for you,” (3) “Using the Internet to chat with other smokers who are trying to quit,” (4) “Receiving emails timed around your quit date that contain professional advice about quitting that is customized for you,” (5) “Attending a program led by a professional and that involves a few meetings with other smokers trying to quit,” (6) “Using nicotine replacement therapy (eg, the patch) which is available without a prescription,” (7) “Using booklets or other printed materials that give professional advice on how to quit,” (8) “Receiving text messages on your cell phone timed around your quit date that contain professional advice about quitting that is customized for you,” and (9) “Using prescription medications for quitting such as Zyban (bupropion) or Chantix (varenicline).”

## Results

### Descriptive Statistics

The majority of respondents had tried to quit in the past year (730, 63.2%) (see Table 1). In addition, 22.2% (266) of smokers planned to quit immediately (ie, in the next 24 hours), 30.3% (362) in the next week or two, 17.5% (209) in the next month, 9.5% (114) in 6 months, and 20.5% (245) at some undecided time in the future.

A large majority of smokers (861, 74.7%) did not know whether free help from a quitline counselor was available to all smokers in their state; 3.0% (35) indicated that such help was *not* available and 21.4% (256) indicated that it was. Not surprisingly, a similar majority, 76.6% (884), reported not ever having used a quitline, 9.7% (112) reported previously using a quitline, and 13.7% (158) indicated this question was not applicable because they had never before tried to quit.

The cessation method that received the greatest proportion of respondents who indicated being “quite a bit” or “very much” interested was NRT (572/1196, 47.8%), followed by a website that provides customized quitting advice (534/1196, 44.6%) and prescription medications (447/1196, 37.4%). Only 11.5% (138/1196) of respondents indicated being “quite a bit” or “very much” interested in using quitlines, and only 17.4% (208/1196) reported similar interest in receiving customized text messages. The proportion of respondents in each sociodemographic category who were “quite a bit” or “very much” interested in a particular cessation method are presented in Table 2. For example, among respondents 25 years or younger (n = 164), only 8.5% (14) were “quite a bit” or “very much” interested in quitlines whereas 39.6% (65) had a similarly strong interest in NRT.

**Table 2:** Number and percentage of respondents in each sociodemographic category who were “quite a bit” or “very much” interested in each cessation method

		Quitline	Website	Web peer support	Emails	Group programs	NRT^a^	Prescription medications	Cessation booklets	Text messages	n
**Age group (years)**											
	≤25	14 (8.5%)	64 (39.0%)	33 (20.1%)	47 (28.7%)	30 (18.3%)	65 (39.6%)	51 (31.1%)	34 (20.7%)	45 (27.4%)	164
	26–40	44 (10.0%)	94 (43.2%)	94 (21.5%)	162 (37.0%)	85 (19.4%)	217 (49.5%)	187 (42.7%)	146 (33.3%)	79 (18.0%)	438
	41–55	54 (15.1%)	87 (50.8%)	87 (24.3%)	143 (39.9%)	94 (26.3%)	185 (51.7%)	138 (38.5%)	121 (33.8%)	52 (14.5%)	358
	≥56	9 (10.5%)	21 (50.0%)	21 (24.4%)	35 (40.7%)	24 (27.9%)	47 (54.7%)	30 (34.9%)	28 (32.6%)	10 (11.6%)	86
**Gender**											
	Male	24 (8.2%)	120 (41.2%)	50 (17.2%)	90 (30.9%)	56 (19.2%)	142 (48.8%)	93 (32.0%)	71 (24.4%)	44 (15.1%)	291
	Female	106 (12.6%)	391 (46.5%)	200 (23.8%)	324 (38.6%)	193 (23.0%)	411 (48.9%)	338 (40.2%)	284 (33.8%)	154 (18.3%)	840
**Education**											
	Grade ≤11	10 (17.2%)	23 (39.7%)	15 (25.9%)	21 (36.2%)	9 (15.5%)	17 (29.3%)	17 (29.3%)	17 (29.3%)	9 (15.5%)	58
	High school graduate/GED^b^	24 (10.0%)	103 (43.1)	53 (22.2%)	82 (34.3%)	47 (19.7%)	122 (51.0%)	87 (36.4%)	65 (27.2%)	30 (12.6%)	239
	Some college	46 (10.2%)	196 (43.7%)	99 (22.0%)	157 (35.0%)	99 (22.0%)	226 (50.3%)	184 (41.0%)	154 (34.3%)	79 (17.6%)	449
	College graduate or higher	51 (13.1%)	190 (49.0%)	84 (21.6%)	155 (39.9%)	95 (24.5%)	189 (48.7%)	144 (37.1%)	120 (30.9%)	81 (20.9%)	388
**Race**											
	White	105 (10.7%)	452 (45.9%)	210 (21.3%)	359 (36.5%)	206 (20.9%)	490 (49.8%)	376 (38.2%)	303 (30.8%)	164 (16.7%)	984
	Black/African American	15 (26.3%)	28 (49.1%)	18 (31.6%)	28 (49.1%)	22 (38.6%)	26 (45.6%)	20 (35.1%)	24 (42.1%)	18 (31.6%)	57
	Asian	2 (11.1%)	8 (44.4%)	5 (27.8%)	5 (27.8%)	4 (22.2%)	8 (44.4%)	8 (44.4%)	8 (44.4%)	4 (22.2%)	18
	Other	9 (12.5%)	23 (31.9%)	16 (22.2%)	23 (31.9%)	17 (23.6%)	28 (38.9%)	27 (37.5%)	21 (29.2%)	13 (18.1%)	72

^a^ Nicotine replacement therapy.

^b^ General equivalency diploma.

### Cluster Analysis

**Figure 1:**
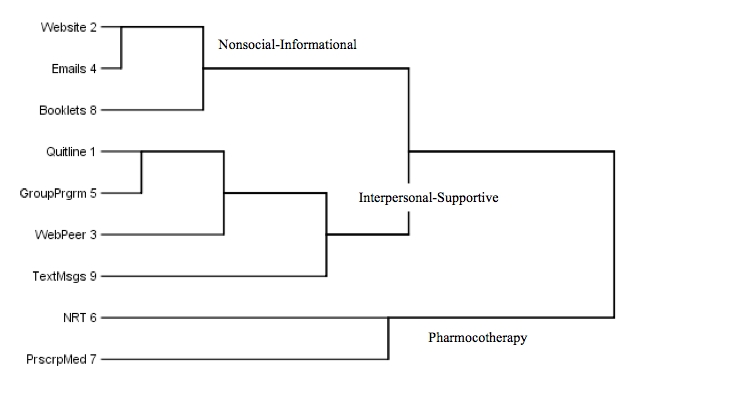
Dendrogram indicating clusters from cluster analysis of interest in cessation products and services (Msgs = messages, NRT = nicotine replacement therapy, Prgrm = program, PrscrpMed = prescription medication)

Inspection of the dendrogram from the cluster analysis and membership at each cluster stage suggested that a 3-cluster solution was appropriate (Figure 1). Of the 3 clusters, one consisted of methods that all involved interpersonal interaction or support, specifically using a telephone quitline, attending a group-based cessation program, using the Internet to chat with other smokers who are trying to quit, and receiving text messages by cell phone. This group of cessation products and services were labeled *interpersonal* *–* *supportive* methods. The appearance of text messages in this cluster is likely attributable to the fact that text messages are typically used for interpersonal reasons—for example, to communicate among friends.

A second cluster consisted of using a customized website, receiving timed emails, and using printed materials that give professional advice on how to quit. We labeled this group of treatments *nonsocial* *–* *informational* methods. The third and final cluster consisted of using NRT and prescription medications. We labeled this cluster *pharmacotherapy*.

Composite variables to represent interest in each of the 3 types of cessation methods were computed as the average of the variables in each cluster. The means and standard deviations for each cluster were as follows: nonsocial–informational methods (mean 2.9, SD 1.2); interpersonal–supportive methods (mean 2.1, SD 1.0); and pharmacotherapy (mean 3.0, SD 1.3). Paired samples *t* tests indicated that interest in interpersonal–supportive methods was significantly lower than interest in pharmacotherapy (t_1195_ = –21.64, *P* < .001) or interest in nonsocial–informational methods (t_1195_ = 30.03, *P* < .001). Interest in nonsocial–informational methods and in pharmacotherapy were only marginally different (t_1195_ = –1.72, *P* = .09). Only 5% (60) of smokers were “quite a bit” or “very much” interested in interpersonal–supportive methods compared to 25% (298) for nonsocial–informational methods and 33% (399) for pharmacotherapy.

Results from multivariate analyses are reported in Table 3. Analyses that controlled for the number of days between when the survey was completed and the start date of the survey revealed the same pattern of significance for multivariate and univariate associations. Univariate analyses indicated that all independent variables except race and education were associated with interest in pharmacotherapy. Also, all independent variables except age were associated with interest in interpersonal–supportive methods. Only quit date was significantly associated with interest in nonsocial–informational methods (*F*_4,938_ = 8.10, *P* < .001). Age group showed a marginal association (*F*_3,938_ = 2.50, *P* = .06).

**Table 3:** Results of multivariate analysis of variance indicating main effects of independent variables on smokers’ interest in cessation treatments

Multivariate			Univariate			
Independent variable	Pillai trace	*P*^a^	Dependent variable	*F*	df	*P*^b^
Quit date	4.21	<.001	Pharmacotherapy	4.11	4, 938	.003
			Interpersonal–supportive	4.04	4, 938	.003
			Nonsocial–informational	8.10	4, 938	<.001
Used a quitline before	3.79	<.001	Pharmacotherapy	4.18	2, 938	.016
			Interpersonal–supportive	8.69	2, 938	<.001
			Nonsocial–informational	1.26	2, 938	.28
Smoking rate	1.95	.04	Pharmacotherapy	3.02	3, 938	.03
			Interpersonal–supportive	2.74	3, 938	.04
			Nonsocial–informational	1.48	3, 938	.22
Age group	2.48	.008	Pharmacotherapy	3.30	3, 938	.02
			Interpersonal–supportive	0.31	3, 938	.82
			Nonsocial–informational	2.50	3, 938	.06
Race	2.59	.006	Pharmacotherapy	0.31	3, 938	.82
			Interpersonal–supportive	6.71	3, 938	<.001
			Nonsocial–informational	1.64	3, 938	.18
Education level	2.16	.02	Pharmacotherapy	2.15	3, 938	.09
			Interpersonal–supportive	3.37	3, 938	.02
			Nonsocial–informational	0.84	3, 938	.47

^a^ Indicates significance of multivariate relationship between the independent variable and the set of 3 dependent variables.

^b^ Indicates significance of univariate relationship between the independent variable and each dependent variable.

### Quit date

The later smokers planned to quit, the greater their interest in pharmacotherapy. While 48% (69/143) of smokers planning to quit within a day were “quite a bit” or “very much” interested in pharmacotherapy, a significantly greater proportion (≥75%); planning to quit later were similarly interested, either “in the next week or two” (132/175, 75%; n = 318, χ^2^_1_ = 25.0, *P* < .001, *V* = .28), or “in the next month” (76/97, 78%; n = 240, χ^2^_1_ = 21.9, *P* < .001, *V* = .30). In contrast, interest in interpersonal–supportive methods was greater the earlier smokers planned to quit; over twice as many smokers planning to quit “in the next week or two” (22/82, 27%) were “quite a bit” or “very much” interested in interpersonal–supportive methods as smokers who planned to quit at some undecided time in the future (8/66, 12%; n = 148, χ^2^_1_ = 4.9, *P* < .02, *V* = .18).

Interest in nonsocial–informational methods was greater among smokers planning to quit in the next week or two (121/144, 84%) compared with those quitting in the next 24 hours (72/107, 67%; n = 251, χ^2^_1_ = 9.7, *P* < .002, *V* = .20). On the other hand, the proportion of smokers planning to quit at some undecided time in the future who were “quite a bit” or “very much” interested was significantly lower (28/71, 39%) than the 67% (72/107) of smokers planning to quit in the next 24 hours (n = 178, χ^2^_1_ = 13.4, *P* < .001, *V* = .28), the 84% (121/144) of smokers planning to quit in the next week or two (n = 215, χ^2^_1_ = 44.4, *P* < .0001, *V* = .46), the 77% (53/69) planning to quit in the next month (n = 140, χ^2^_1_ = 20.0, *P* < .0001, *V =* .38), or the 75% (24/32) planning to quit in the next 6 months (n = 103, χ^2^_1_ = 11.2, *P* < .001, *V =* .33).

### Previous Use of a Quitline

A greater proportion of smokers who had used a quitline before were “quite a bit” or “very much” interested in pharmacotherapy (49/63, 78%), than the proportion who had never before used a quitline (284/439, 65%; n = 502, χ^2^_1_ = 4.2, *P* = .03, *V =* .09). In addition, smokers who had used a quitline before were much more likely to be “quite a bit” or “very much” interested in interpersonal–supportive methods (13/25, 52%) than smokers who had never before used a quitline (38/234, 16%; n = 259, χ^2^_1_ = 18.3, *P* < .001, *V =* .27) or smokers for whom the question was not applicable (because they had never before tried to quit) (9/33, 27%; n = 58, χ^2^_1_ = 3.7, *P* = .05, *V =* .25).

### Cigarettes Per Day

Interest in pharmacotherapy was significantly associated with smoking level. Among lighter smokers (<10 cigarettes per day), 57% (63/111) were “quite a bit” or “very much” interested in pharmacotherap*y*, whereas a greater proportion (132/188, 70%) of smokers of between 10 and 19 cigarettes per day (n = 299, χ^2^_1_ = 5.6, *P* = .01, *V =* .14) or between 20 and 29 cigarettes per day (118/164, 72%) were similarly highly interested (n = 275, χ^2^_1_ = 6.7, *P* = .007, *V =* .16). The very heaviest smokers, however, were somewhat less interested in pharmacotherapy than moderate smokers, although the differences were marginally significant. Specifically, 59% (30/51) of participants who smoked 30 or more cigarettes per day were “quite a bit” or “very much” interested in pharmacotherapy, compared with the 70% (132/188) of those smoking between 10 and 19 cigarettes per day (n = 239, χ^2^_1_ = 2.4, *P* = .09, *V =* .10), or the 72% (118/164) of those smoking between 20 and 29 cigarettes per day (n = 215, χ^2^_1_ = 3.1, *P* = .06, *V =* .12).

Although the MANOVA and univariate tests indicated a significant relationship between cigarettes per day and interest in interpersonal–supportive methods, none of the pairwise Tukey test comparisons were significant (all *P* > .05). These results are thus not reported.

### Age Group

A significantly smaller proportion of younger smokers (≤25 years) were “quite a bit” or “very much” interested in pharmacotherapy (51/88, 58%) than the proportion (163/220, 74%) of smokers in the 26 to 40 age group (n = 308, χ^2^_1_ = 7.2, *P* = .005, *V =* .16) who were similarly interested.

The univariate *F* test for the relationship between age group and interest in nonsocial–informational methods was marginally significant (*P* < .06) but Tukey pairwise comparisons indicated significant differences between age groups (all *P* < .05) that were corroborated by chi-square analyses. Fewer younger smokers (≤25 years) were “quite a bit” or “very much” interested in nonsocial–informational methods (32/56, 57%) than all other age groups: 74% (111/151) of smokers 26–40 years (n = 207, χ^2^_1_ = 5.1, *P* = .02, *V =* .16), 79% (102/130) of smokers 41–55 years (n = 186, χ^2^_1_ = 8.8, *P* = .003, *V =* .22), and 83% (25/30) of smokers aged ≥56 years (n = 86, χ^2^_1_ = 6.0, *P* = .01, *V =* .26).

### Race

White and black/African American smokers differed in their interest in interpersonal–supportive methods. While only 18% (44/242) of white smokers were “quite a bit” or “very much” interested in interpersonal–supportive methods, a much greater percentage (7/15, 47%) of black/African American smokers were similarly interested (n = 257, χ^2^_1_ = 7.2, *P* = .01, *V =* .17). These results should be interpreted cautiously, however, due to the small sample of African American smokers contributing to these analyses.

### Education

Although the univariate *F* test for the relationship between education level and interest in pharmacotherapy was marginally significant (*P* < .10), Tukey pairwise comparisons indicated significant differences between education levels that were corroborated by chi-square analyses. These indicated that a smaller proportion of smokers who had completed grade 11 or less were “quite a bit” or “very much” interested in pharmacotherapy (12/34, 35%) than the proportion of smokers who had achieved higher levels of education; specifically high school or its equivalent (87/122, 71%; n = 156, χ^2^_1_ = 14.9, *P* < .001, *V =* .31), some college (161/237, 68%; n = 271, χ^2^_1_ = 13.7, *P* < .001, *V =* .23), or graduation from college or higher (124/182, 68%; n = 216, χ^2^_1_ = 13.2, *P* < .001, *V =* .25).

## Discussion

Increasing the demand for cessation products and services will lead to more quit attempts, higher cessation rates, and greater reductions in smoking prevalence [22]. Smokers who seek cessation information on the Internet are a large group of smokers [11] who can be targeted and exposed to online messages that could potentially increase demand for evidence-based cessation products. Little is known, however, about what these smokers need or want to help them quit, or the reasons for or predictors of their preferences for various treatments. The current study asked smokers who visited the website of the ACS’s GASO for cessation information to rate their level of interest in evidence-based cessation products and services. Our results for predictors of interest in cessation methods were obtained controlling for all other variables including gender, knowledge of availability of free quitlines, and past-year attempts.

Cluster analysis of smokers’ ratings suggested that smokers’ interest in behavioral treatments centered on the degree of social or interpersonal involvement or social support the treatment would provide. Specifically, one cluster we obtained comprised products and services that involve high levels of interpersonal interaction and/or support. These included group-support cessation programs, telephone counseling, and using the Internet to chat with other smokers trying to quit. A contrasting cluster consisted of cessation methods that would provide tailored or individualized information on cessation, but that would *not* involve interpersonal interaction. The latter comprised tailored emails timed around a quit date, a website providing tailored cessation information, or booklets with information on quitting. Among the current sample of smokers, these nonsocial–informational programs were preferred to a significantly greater degree than methods requiring interpersonal interaction or support.

A separate cluster was also obtained that, as hypothesized, consisted of pharmacotherapies for cessation. This result suggests that motivation to use pharmacotherapy extends to both nicotinic and non-nicotinic medications. We had also hypothesized that interest in pharmacotherapy would be greater than interest in behavioral methods. We found, however, that smokers’ interest in medications as a whole was comparable with their interest in nonsocial–informational methods. Interest in interpersonal–supportive methods received lower ratings than pharmacotherapy or nonsocial–informational methods. These results are consistent with Cobb and Graham’s [11] finding that Internet seekers of cessation information believed that tailored information would be more helpful than telephone counseling or support received through Internet chat or forums. The present study builds on their findings by (1) suggesting that the level of interpersonal interaction and social support involved in these treatments makes them less appealing to Internet seekers of cessation information, and (2) examining sociodemographic and other factors associated with interest in interpersonal–supportive, nonsocial–informational, and pharmacological methods for cessation.

As noted earlier, compared with traditional media such as radio or television advertising, online advertising has been shown to be a more cost-efficient mode of recruiting smokers to Internet and telephone-based cessation treatments [12]. Moreover, behavioral targeting can be conducted whereby marketers target online consumers who fit particular sociodemographic criteria based on histories of websites visited and other publicly available information. These strategies can be used to promote cessation products and services to smokers seeking assistance via the Internet. Our results suggest, however, that this is more likely to be successful if the focus is on nonsocial–informational methods and/or pharmacotherapy. The low interest in interpersonal–supportive methods among online seekers of cessation information, however, suggests that methods involving interpersonal interaction and/or support would need to be carefully marketed. They should also consider the various smoker characteristics associated with greater interest in these methods.

### Pharmacotherapy

Our results indicated that more smokers who were quitting the next day were not at all interested in pharmacotherapy compared with smokers quitting later on. Smokers planning to quit the next day most likely have made up their minds about quitting right away. They therefore may not want to spend the time to learn about and choose a medication, go to the drugstore, or ask a doctor to write a prescription. However, given the efficacy of pharmacotherapy, efforts might be aimed at these smokers to set a quit date with enough time to consider pharmacotherapy.

As hypothesized, results indicated that a greater proportion of older smokers (26–40 years) than younger smokers (≤25 years) were strongly interested in pharmacotherapy. This suggests that older smokers would be more receptive than younger smokers to efforts that encourage pharmacotherapy use. It is not immediately clear why a smaller proportion of younger smokers were interested, but one possibility is that more of them may believe they cannot afford medications. Alternatively, younger smokers may hold myths about medications that older smokers know are not true. Another possibility is that older smokers are more accustomed to taking medications for various health conditions. These possibilities can be examined in future research addressing preferences for cessation methods. The affordability of medications, or myths about them, may also explain why a much smaller proportion of less educated smokers were interested in pharmacotherapy than more educated smokers. Understanding the main reasons for less educated smokers’ reluctance to use pharmacotherapy through further research would be important in devising strategies to increase demand for its use in this population.

Strategies to increase demand for pharmacotherapy should also consider smoking level, as the number of cigarettes smoked per day was associated with interest in pharmacotherapy. Fewer of the lightest smokers (defined as smoking <10 cigarettes per day) and fewer of the heaviest smokers (≥30 cigarettes per day) were interested in pharmacotherapy than were those who smoked between 10 and 29 cigarettes per day. A possible explanation is that lighter smokers may be more likely to believe that they are not so addicted that they need pharmacotherapy to help them quit. In contrast, the heaviest smokers may be more likely to believe that they are so addicted that even medications cannot help them quit. If evidence is obtained supporting these reasons, they can be addressed in online messages to increase demand for pharmacotherapy such as NRT.

Based on the results obtained, behavioral marketing could potentially be used to increase demand for NRT by targeting smokers 26–40 years old who have at least a high school education (as these individuals appear to be most interested in pharmacotherapy). Messages for these individuals could emphasize the ease of purchasing NRT, compare its cost relative to continued smoking, clarify concerns about using nicotine for treatment, and note its effectiveness relative to no medications. Focus-group research would be helpful in determining the precise content of messages that would resonate most for this demographic segment.

### Interpersonal–Supportive Methods

In general, smokers were less interested in interpersonal–supportive cessation methods than in pharmacotherapy or nonsocial–informational methods. Moreover, interest in interpersonal–supportive methods could not be explained by whether smokers knew that free quitlines were available (as results were no different when this variable was controlled). In spite of the generally lower interest in interpersonal–supportive methods, however, results indicated that there were differences in interest as a function of quit date and whether smokers had previously used a quitline. Specifically, more smokers planning to quit “in the next week or two” were interested in interpersonal–supportive methods than were smokers who planned to quit at some undecided time in the future. This could be interpreted as a function of the greater seriousness about quitting among smokers planning to quit in the next week or two than among smokers who simply report planning to quit at some undecided time in the future.

Smokers who had previously used a quitline were also much more likely to be interested in interpersonal–supportive methods than smokers who had never before called a quitline. Smokers who had previously used a quitline may have had a positive enough experience to consider using an interpersonal–supportive method again. Alternatively, these methods may appeal to these smokers because of preexisting personal characteristics. For example, smokers higher on the personality trait of extraversion may find social interactional methods more appealing.

We had hypothesized that male smokers would be less interested than female smokers in interpersonal–supportive methods. Gender, however, was unrelated to interest in these treatments or, for that matter, in the two other types of treatments. Previous research has found gender differences among young adult smokers in the seeking of help from a professional [15]. Female smokers are also more frequent users of quitline services [14]. The lack of gender differences in *interest* in interpersonal– supportive methods in this study suggests that there may be other reasons why men have not actually used these methods as frequently as women. One possibility is that men may not be as knowledgeable about their existence. In this study, however, men and women did not differ in their knowledge about the availability of free quitlines. Alternatively, perhaps men are intrinsically interested in interpersonal–supportive methods but choose to not use them because of concerns about conforming to gender stereotypes of masculinity and help-seeking. Future research testing this hypothesis would be useful. Support for this hypothesis would suggest that online marketing messages directed to male smokers that also address the issue of masculinity might be able to encourage their greater use of interpersonal–supportive methods.

Overall, these results indicate that it may take a greater amount of effort to persuade smokers using the Internet for cessation advice to use interpersonal–supportive methods such as quitlines, group counseling, or Internet forums for peer support. However, given the demonstrated efficacy of quitlines, efforts should be made to encourage these options, at least among those smokers who would be more receptive (ie, smokers planning to quit earlier and who may have used a quitline before).

### Nonsocial–Informational Methods

Nonsocial–informational methods include Internet-based treatments for tobacco use, which reviews and meta-analyses have concluded are effective compared with minimal or no treatments [23,24]. These and other Internet-based methods are typically free. Moreover, they can affect prevalence at the population level because large numbers of smokers can be reached for a very low cost.

Only age and when smokers were planning to quit were significantly related to interest in nonsocial–informational methods. While among all age groups most were “quite a bit” or “very much” interested in these methods, a greater majority of *older* smokers expressed strong interest. This counters the perception that older individuals are not receptive to newer technologies such as Web-delivered treatments or tailored email-based cessation programs. At the time that smokers are looking for cessation information on the Internet it seems logical to promote and provide access to these methods. Such a strategy may be most effective if targeted toward older smokers and to smokers who are a week or two away from their planned quit date.

### Limitations

The sample of individuals in this study may not be representative of the population of smokers who use the Internet for cessation advice. Nevertheless, some of our results for interest in cessation methods parallel actual use found by studies using different recruitment methods. For example, adult smokers in the 2005 NHIS reported a low rate of having used behavioral treatments (which included in-person or telephone counseling or group cessation programs) compared with pharmacotherapy. This is consistent with the current study’s finding of greater interest in pharmacotherapy than in behavioral methods. The NHIS also found that young adult smokers were less likely than older smokers to use pharmacotherapy [14]. Consistent with this we found that a significantly greater proportion of older smokers than younger smokers were “quite a bit” or “very much” interested in using pharmacotherapy. In addition, our results are similar to Cobb and Graham’s finding that a greater proportion of Internet seekers of cessation information were women [11]. That study also found that telephone counseling and support received through Internet chat or forums were perceived as treatments that would not be helpful. In addition, although Cobb and Graham did not assess education levels, the higher levels of education noted in the current sample is consistent with that of users of the Internet in general [25], the population of interest in the current study. Nevertheless, replication of our findings would add confidence to our results for smokers’ preferences and factors associated with them. In addition, our finding suggesting that black smokers were more interested than white smokers in interpersonal–supportive methods for quitting should be further investigated. Replication of this result would justify devoting extra resources to encourage the use of quitlines and other interpersonal–supportive methods among smokers from this community.

It is also not certain whether interest in a type of cessation method would translate into actual use of that method if it were made available. The aim of the study, however, was not to predict actual use but rather to first understand the basis of smokers’ preferences. This is information that can then be used to increase adoption of evidenced-based treatments. Self-report is a reasonable approach to understanding these preferences and is typically a first step in market research. Future research may then focus on how to increase demand based on knowledge of these preferences by developing appropriate messaging for particular segments of consumers. Making preferred treatments appealing and available at the time smokers are seeking cessation advice on the Internet would help translate preferences to actual behavior, especially given the convenience of the Internet for purchasing products and services. There is also no reason to believe that ratings of preferences for particular cessation methods would be subject to social desirability biases. In the case of tobacco use behavior, self-reports have been shown to be reliable and valid [26], including from online surveys [27]. The validity of self-reports of tobacco behavior in online surveys is attributed to the lack of incentive to present oneself in a favorable light. In contrast, in a clinical setting where smokers are face to face with researchers, social desirability biases are greater [26]. In the current study, smokers planning to quit completed an anonymous survey with no incentives.

Due to concerns for brevity, the study was unable to test specific underlying reasons for associations between predictors and interest in treatments. For example, why a greater proportion of older smokers were strongly interested in nonsocial–informational cessation methods than were younger smokers could not be answered by the present study. Understanding smokers’ reasons behind their interest (or noninterest) in cessation methods would be a fruitful topic for further research. Results of that research would help in refining strategies to promote use of cessation products and services for all sociodemographic groups. Evaluating smokers’ interest in cessation aids with no evidence of effectiveness (eg, hypnosis, acupuncture) would also be informative.

### Conclusions

Smokers who want to quit have available to them several effective cessation products and services. Demand for these is relatively low, however. Fortunately, many smokers access the Internet to help them quit, so targeting these smokers to promote cessation aids is a potentially effective way of increasing demand. Results of the current study indicated that they have relatively greater interest in pharmacotherapy and in cessation methods that provide tailored information or advice, but that do *not* involve interpersonal or socially supportive interactions. Moreover, the study indicated that smokers’ level of cigarette consumption, when they were planning to quit, and sociodemographic variables were all associated with level of interest in using these treatments. Future research investigating the causes of interest in evidence-based treatments and whether targeted messages can encourage use among different groups of smokers will be an important step in understanding how to increase demand for treatments among smokers who are Internet users.

## Abbreviations

ACS: American Cancer Society
GASO: Great American Smokeout
MANOVA: multivariate analysis of variance
NHIS: National Health Interview Survey
NRT: nicotine replacement therapy
